# Direct and negative regulation of the *sycO-ypkA-ypoJ *operon by cyclic AMP receptor protein (CRP) in *Yersinia pestis*

**DOI:** 10.1186/1471-2180-9-178

**Published:** 2009-08-25

**Authors:** Lingjun Zhan, Lei Yang, Lei Zhou, Yingli Li, He Gao, Zhaobiao Guo, Lianfeng Zhang, Chuan Qin, Dongsheng Zhou, Ruifu Yang

**Affiliations:** 1 State Key Laboratory of Pathogen and Biosecurity, Beijing Institute of Microbiology and Epidemiology, Beijing 100071, PR China; 2 Institute of Laboratory Animal Sciences, Chinese Academy of Medical Peking Union Medical College, Beijing, 100021, PR China

## Abstract

**Background:**

Pathogenic yersiniae, including *Y. pestis*, share a type III secretion system (T3SS) that is composed of a secretion machinery, a set of translocation proteins, a control system, and six Yop effector proteins including YpkA and YopJ. The cyclic AMP receptor protein (CRP), a global regulator, was recently found to regulate the laterally acquired genes (*pla *and *pst*) in *Y. pestis*. The regulation of T3SS components by CRP is unknown.

**Results:**

The *sycO*, *ypkA *and *yopJ *genes constitute a single operon in *Y. pestis*. CRP specifically binds to the promoter-proximate region of *sycO*, and represses the expression of the *sycO-ypkA-yopJ *operon. A single CRP-dependent promoter is employed for the *sycO-ypkA-yopJ *operon, but two CRP binding sites (site 1 and site 2) are detected within the promoter region. A CRP box homologue is found in site 1 other than site 2. The determination of CRP-binding sites, transcription start site and core promoter element (-10 and -35 regions) promotes us to depict the structural organization of CRP-dependent promoter, giving a map of CRP-promoter DNA interaction for *sycO-ypkA-yopJ*.

**Conclusion:**

The *sycO-ypkA-yopJ *operon is under the direct and negative regulation of CRP in *Y. pestis*. The *sycO-ypkA-yopJ *promoter-proximate regions are extremely conserved in *Y. pestis*, *Y. pseudotuberculosis *and *Y. enterocolitica*. Therefore, data presented here can be generally applied to the above three pathogenic yersiniae.

## Background

Plague, caused by *Yesinia pestis*, is a zoonotic disease that threatened public health seriously. The three pathogenic *Yersinia *species, *Y. pestis*, *Y. pseudotuberculosis*, and *Y. enterocolitica*, share a type III secretion system (T3SS) that is composed of a secretion machinery, a set of translocation proteins, a control system, and six Yop effector proteins [1,2]. Through the T3SS, pathogenic yersiniae inject effectors into the cytosol of eukaryotic cells when docking at the surface of host cell. The injected Yops perturb the signaling cascades that activate the processes of phagocytosis, cytokine release and respiratory burst. As a result, phagocytosis is inhibited, recruitment of PMNs and monocyte-derived macrophages is reduced, and lymphocyte proliferation is prevented.

The cyclic AMP receptor protein (CRP) is a global regulator that controls the transcription initiation for more than 100 bacterial genes/operons [3]. CRP is activated by cyclic AMP (cAMP), forming the cAMP-CRP complex. This complex binds a symmetrical consensus DNA sequence TGTGA-N_6_-TCACA (known as the CRP box sequence) located within the upstream promoter regions. The CRP-promoter DNA interaction is crucial for the regulation of target genes.

CRP and its homologues are required for virulence and/or expression of virulence genes in several pathogens, including *Y. pestis *[4], *Y. enterocolitica *[5], *Vibrio vulnificus *[6], *Vibrio cholerae *[7] and *Mycobacterium tuberculosis *[8]. The *crp *disruption in *Y. pestis *attenuates both *in vitro *and *in vivo *growth of the mutant, and leads to a >15,000-fold loss of virulence after subcutaneous infection, but a less than 40-fold increase in LD50 by intravenous inoculation [4]. CRP plays a role in the globally transcriptional regulation of genes including a wide set of virulence genes in *Y. pestis *[4]. Especially, it directly stimulates the expression of plasminogen activator (Pla) [4,9], a virulence factor essential for bubonic and primary pneumonic plague [10,11].

*Yersinia *protein kinase A (YpkA) and *Yersinia *outer protein J (YopJ) are encoded by plasmid pCD1-borne *ypkA *and *yopJ *genes in *Y. pestis*, respectively. YpkA/YopO is a serine/threonine protein kinase involved in host actin cytoskeletal rearrangements and in inhibition of phagocytosis [12], while YopJ/YopP acts as an acetyltransferase inhibiting mitogen-activated protein kinase (MAPK) and the nuclear factor kappaB (NFκB) signaling pathways used in innate immune response [13]. Both of them are the effector proteins of T3SS and essentially contribute to the virulence of *Y. pestis *[2,14]. SycO is a T3SS chaperone that increases solubility and secretion efficiency of the effector YpkA/YopO [15].

In the present work, we disclosed that CRP directly and negatively regulated the *sycO-ypkA-yopJ *operon in *Y. pestis *under the calcium-rich condition, by using real-time RT-PCR, *LacZ *reporter fusion, electrophoretic mobility shift assay (EMSA), and DNase I footprinting assay. Data presented here further validated the important role of CRP in virulence of *Y. pestis*.

## Methods

### Bacterial strains

The wild-type (WT) *Y. pestis *strain 201 belongs to a newly established *Y. pestis *biovar, *Microtus *[16], which was thought to be avirulent to humans, but highly virulent to mice. An in-frame deletion of the *crp *gene was constructed by using one step inactivation method [17], generating a mutant strain referred to as *Δcrp *[4]. Bacteria were grown in Luria-Bertani (LB) broth or chemically defined TMH medium [18] at 26 or 37°C. *E. coli *was grown in LB broth at 37°C. When needed, antibiotics were added at the following concentrations: 100 μg/ml for ampicillin, 50 μg/ml for kanamycin, and 34 μg/ml chloramphenicol.

### Bacterial growth and RNA isolation

The WT and *Δcrp *were grown at 26°C in the TMH medium with the addition of 1 mM cAMP (referred to as 'TMH-1mM cAMP') to an OD_620 _of about 1.0, and then diluted by 20-fold into the fresh 'TMH-1mM cAMP' medium for cultivating at 26°C until an OD_620 _of about 1.0, and finally transferred to 37°C for 3 h. Bacterial cells were harvested for the isolation of total RNA. Immediately before harvesting, bacterial cultures were mixed with RNAprotect Bacteria Reagent (Qiagen) to minimize RNA degradation. Total RNA was isolated using the MasterPure™ RNA Purification kit (Epicenter). Contaminated DNA in RNA samples was removed by using the Amibion's DNA-free™ Kit. RNA quality was monitored by agarose gel electrophoresis and RNA quantity was measured by spectrophotometer.

### Real-time RT-PCR

Gene-specific primers (Table 1) were designed to produce a 150 to 200 bp amplicon for each gene. cDNAs were generated by using 5 μg of RNA and 3 μg of random hexamer primers. Using three independent cultures and RNA preparations, real-time PCR was performed in triplicate as described previously [4], through the LightCycler system (Roche) together with the SYBR Green master mix. Based on the standard curve of 16S rRNA expression for each RNA preparation, the relative mRNA level was determined by the classic ΔCt method. 16S rRNA gene was used to normalize that of all the other genes. The transcriptional variation between the WT and *Δcrp *strains was then calculated for each gene. A mean ratio of two was taken as the cutoff of statistical significance.

**Table 1:** Oligonucleotide primers used in this study

Target gene	Primer sequence (5'→3')
**EMSA (Sense/antisense)**	
*sycO*	ATATTCTGGGACGGGTTT/TTCCTGCTGAGTTTCTGC
YPO1099	AGCCCTCTCTCCCTAGCC/GCAGTTGCCAGACCGC
YPO0180	GCTACCGAGCCTAACCC/AGGCACCCATCTCATGG

**Real-time PCR or RT-PCR (Sense/antisense)**	
*sycO*	GCCCTTGTTTCGCTTGGAGTG/AGTTCCTGCTGAGTTTCTGCTG
*ypkA*	GCTAAGATTGAACGCTCCATTG/TCAGAACAACGCCAACCATC
*yopJ*	AATCCAGGCGAACAATAAATATCC/CACTGAAATGTATTCCACCTTCC
*sycO-ypkA *intergenic	CAGGAACTGCCCCTTCATAC/ATACCGTTTTCCTCCGATATTGAG
*ypkA-yopJ *intergenic	TGCGAGAGCTGACGACCATC/TCATTACTGATTAAAGAACTGGTC
*lacA*	CCGATAACGATTGGCAATAACG/GCGAATAACCCGACAAGGAAC
16s rRNA	TTACCTACTCTTGACATCCAC/GCTGGCAACAAAGGATAAG

**DNase I footprinting (Sense/antisense)**	
*sycO*	CAGATTTGTCTACAGGTTCG/CTCAGCATAATAACGACTCGG

**LacZ reporter fusion (Sense/antisense)**	
*sycO*	GCGGAATTCAGGAACGGGAAGATTTAC/GCGGGATCCAATCTCTCTGCATGAACG

**Primer extension**	
*sycO*	CTCAGCATAATAACGACTCGG

### LacZ reporter fusion and β-Galactosidase assay

A 408 bp promoter-proximate of *cycO *(Table 1) was cloned directionally into the *Eco*RI and *Bam*HI sites of plasmid pRS551 expressing LacZ, which was verified by DNA sequencing. The recombinant plasmids were introduced into the WT and *Δcrp*, respectively. The plasmid pRS551 was also transformed as negative control. The resulting strains were grown as described in RNA isolation. β-Galactosidase activity was determined for each strain by using the Promega β-Galactosidase Enzyme Assay System [4]. Assays were performed in triplicate.

### DNA-binding assays

Preparation of purified recombinant His-CRP protein, electrophoretic mobility shift assay (EMSA) and DNase I footprinting assay were conducted as described previously [4]. For EMSA, a 468 bp promoter-proximate region of *cycO *(containing a predicted CRP binding site) or the corresponding cold probe (i.e. unlabeled target DNA) (Table 1) was radioactively labeled, incubated with increasing amounts of purified His-CRP protein, and then subjected to 4% (w/v) polyacrylamide gel electrophoresis. In the DNase I footprinting experiments, coding or noncoding strand (261 bp in length) containing the predicted CRP binding site was labeled with [γ-^32^P] at the 5' end, then, incubated with increasing amounts of His-CRP; after partial digestion with DNase I, the resulting fragments were analyzed by denaturing gel electrophoresis. Radioactive species were detected by autoradiography.

### Primer extension analysis

For the primer extension assay [4], an oligonucleotide primer (Table 1) complementary to a portion of the RNA transcript of each gene was employed to synthesize cDNAs from the RNA templates. Electrophoresis of primer extension products was performed with a 6% polyacrylamide/8M urea gel. The yield of each primer extension product would indicate the mRNA expression level of the corresponding gene in each strain, and further could be employed to map the 5' terminus of RNA transcript for each gene.

## Results

### The *sycO*, *ypkA *and *yopJ *genes constitute a single operon

**Figure 1:**
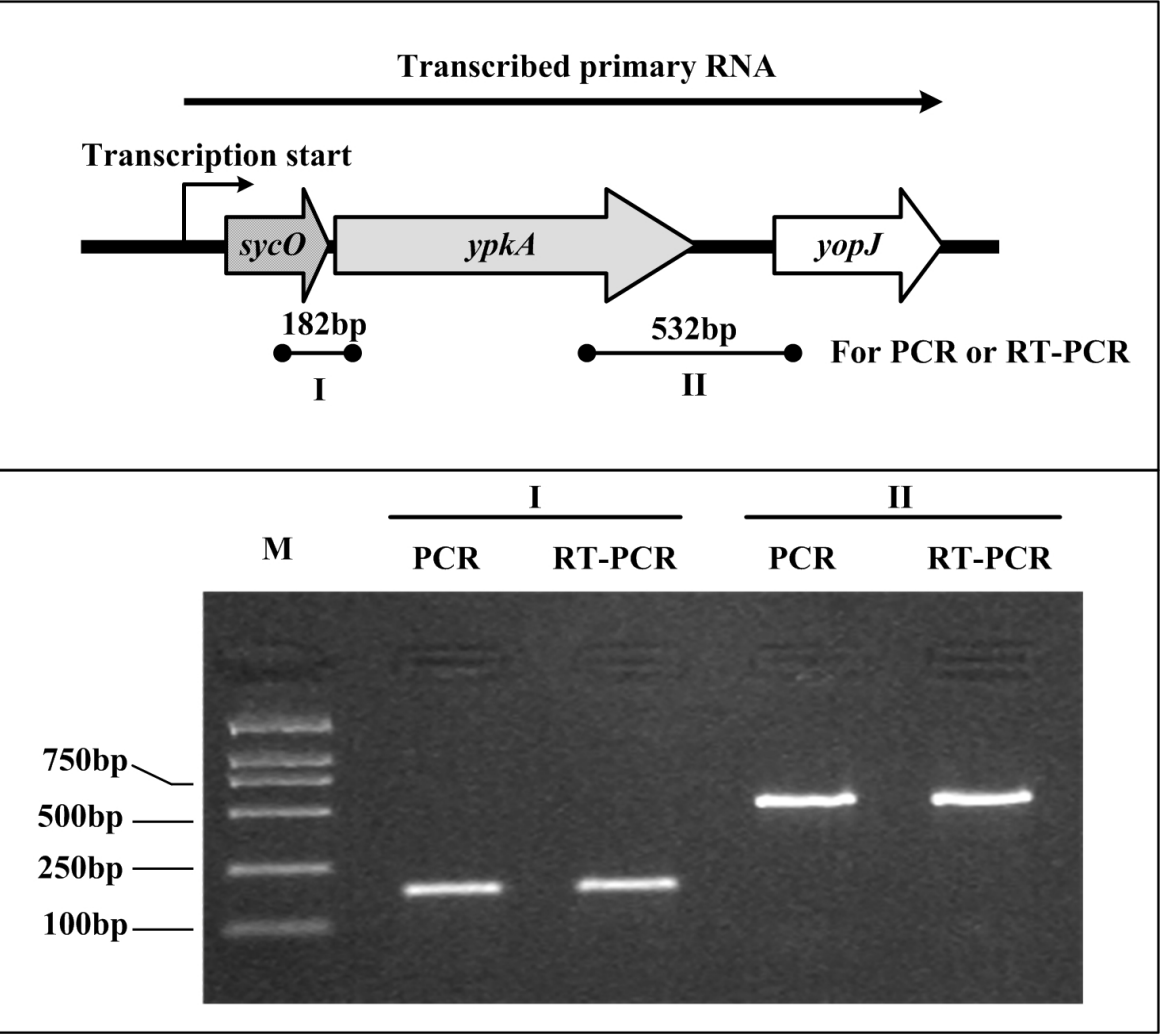
**Transcriptional organization of the *sycO-ypkA-yopJ *operon**. Arrows represent the length and direction of transcription of *sycO*, *ypkA *and *yopJ *on pCD1. The horizontal arrow depicts the putative primary RNA transcript. The arrowheads indicate the location of primer pair and the expected amplicons. Genomic DNA and cDNA generated by RT were used as the templates for PCR and RT-PCR, respectively. To ensure that there was no contamination of genomic DNA in the RT reactions, negative controls of RT-PCR were performed using 'cDNA' generated without reverse transcriptase as templates. Reactions containing primer pairs without template were also included as blank controls. As expected, both negative and blank controls of RT-PCR gave no amplicon (data not shown).

The RT-PCR assay indicated that the *sycO*, *ypkA *and *yopJ *genes (designated as pCD12, pCD13 and pCD14 in *Y. pestis *91001 [19], respectively) were transcribed as a single primary RNA (Fig. 1), and thereby these three genes constituted a single operon in *Y. pestis Microtus *strain 201.

### CRP greatly represses transcription of the *sycO-ypkA-yopJ *operon

**Figure 2:**
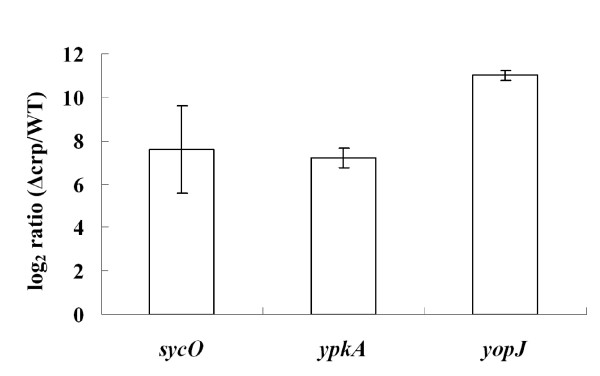
**CRP-dependent transcription of *sycO, ypkA *and *yopJ***. Shown was the mean log_2 _ratio (*Δcrp *versus WT) of mRNA level for each gene.

Our previous cDNA microarray analysis showed that the transcription of *sycO*, *ypkA *and *yopJ *was repressed by CRP [4]. Herein, the real-time RT-PCR assays confirmed that these three genes were up-regulated by more than 50 folds in the *Δcrp *mutant in relative to the WT strain (Fig. 2). Taken together, transcription of the *sycO-ypkA-yopJ *operon was under the negative control of CRP.

### CRP greatly represses promoter activity of *sycO-ypkA-yopJ*

To test the action of CRP on the *sycO-ypkA-yopJ *promoter activity, we constructed the *sycO*::*lacZ *fusion promoter consisting of a 690 bp promoter-proximate region of *sycO *and promoterless *lacZ*, and then transformed into the WT and *Δcrp*, respectively. Empty vector pRS551 was also introduced into them, respectively, as controls. *β*-galactosidase activity was measured for evaluating the *sycO-ypkA-yopJ *promoter activity in each strain. Since the *crp *mutation had an effect on the copy number of recombinant or empty pRS551 plasmid [4], a normalized fold change in the activity of each fusion promoter in WT in relative to *Δcrp *was calculated to avoid the influence of copy number of pRS551 (Table 2).

**Table 2:** Promoter activity determined with the *sycO:lacZ *reporter fusion

	Fold change (*Δcrp*/WT)		Normalized fold change of promoter activity in *Δcrp *in relative to WT
		
*LacZ *fusion	Plasmid copy number	Miller units	
*PsycO-lacZ*	0.006	0.182	30.33

*β*-Galactosidase activity (miller units) was detected as the promoter activity. An extremely low promoter activity was detected for the *Δcrp *or WT transformed with empty pRS551 (data not shown). Copy number of recombinant pRS551 (*PsycO-lacZ*) was determined by real-time quantitative PCR, the detecting fold change of plasmid copy number was set to be 1 to generate a normalization factor that was subsequently used for generating the normalized fold change of promoter activity (miller units) in the *Δcrp *in relative to the WT. Each experiment was done in triplicate.

Accordingly, the *β*-galactosidase activity in the *Δcrp *increased compared to the WT when they grew in the 'TMH-1mM cAMP' medium, indicating that CRP greatly repressed the promoter activity of *sycO-ypkA-yopJ *(Table 2).

### CRP binds to promoter-proximate region of *sycO-ypkA-yopJ*

**Figure 3:**
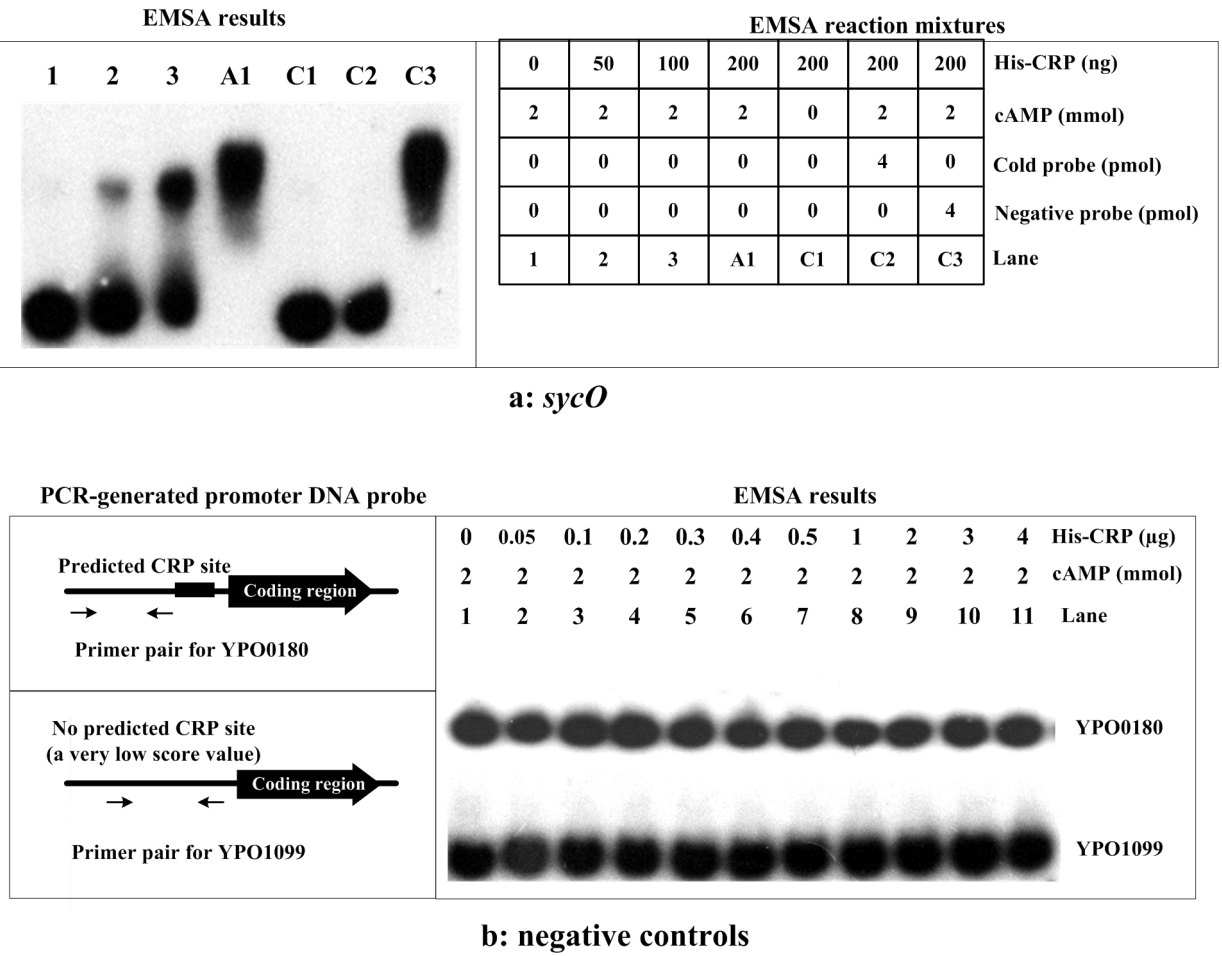
**Electrophoretic mobility shift assay**. The band of DNA fragment containing the promoter region of *sycO *disappeared with increasing amounts of CRP protein, and a retarded DNA band with decreased mobility turned up (Fig. 3a), which presumably represented the CRP-DNA complex. But for YPO0180 and YPO1099, the CRP-DNA complex did not appear even His-CRP was increased to 4 μg for each reaction mixture (Fig. 3b).

A CRP box-like sequence was found in the promoter-proximate region of *sycO-ypkA-yopJ *[4], indicating the direct association of CRP with the *sycO-ypkA-yopJ *promoter region. Further EMSA experiments showed that the cAMP-CRP complex bound to the *sycO-ypkA-yopJ *promoter region in a CRP dose-dependent manner (Fig. 3a). CRP could not bind to the target DNA in the absence of cAMP.

To validate the specificity of CRP-DNA interaction, YPO0180 and YPO1099 [gene IDs in CO92 [20]] were used as negative controls (Fig. 3b). The PCR-generated upstream DNA of YPO0180 did not harbor the predicted CRP binding site, while the YPO1099 upstream region gave an extremely low score value of 0.96 during the pattern matching analysis using the CRP consensus (*sycO *gave a score value of 8.57) [4]. Both of them gave negative EMSA result, even the CRP protein was increased to 4 μg in a single reaction mixture (Fig. 3b).

Therefore, CRP specifically bound to the *sycO-ypkA-yopJ *promoter region and directly repressed the transcription of *sycO-ypkA-yopJ*.

### Structural organization of CRP-dependent *sycO-ypkA-yopJ *promoter

**Figure 4:**
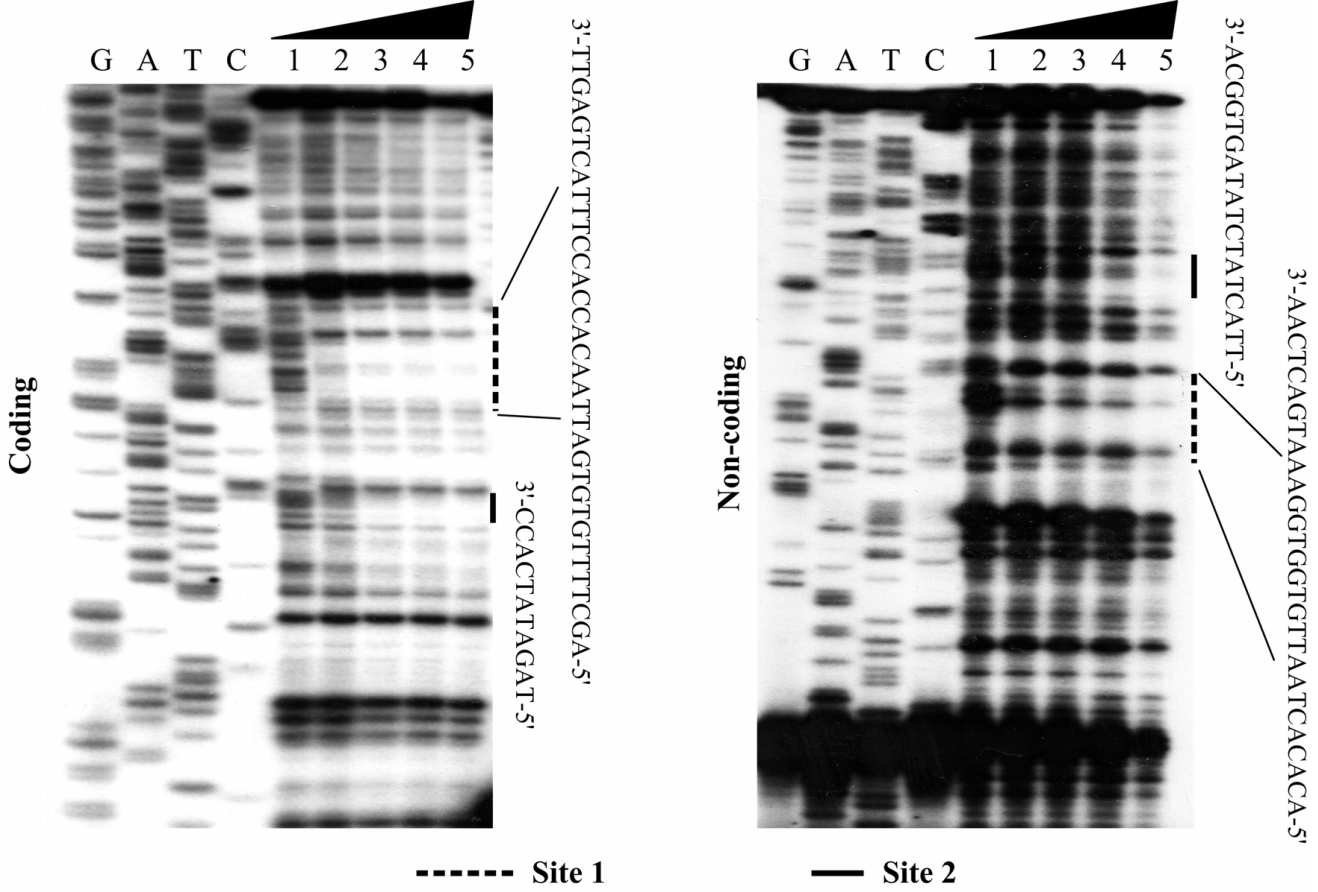
**DNase I footprinting assay**. The labeled DNA probe was incubated with various amounts of purified His-CRP (lanes 1, 2, 3, 4, and 5 contained 0, 500, 1000, 2000 and 3000 ng, respectively), and subjected to DNase I footprinting assay. Lanes G, A, T and C represented the Sanger sequencing reactions. On the right-hand side was indicated the protected regions (bold line). The DNA sequences of footprints were shown from the top (3') to the bottom (5').

In order to locate the precise CRP binding site within the *sycO-ypkA-yopJ *promoter region, DNase I footprinting assay was performed with both coding and non-coding strands. As shown in Fig. 4, CRP protected two distinct DNA regions (sites 1 and 2) against DNase I digestion in a dose-dependent pattern. Only site 1 contained the CRP box-like sequence.

**Figure 5:**
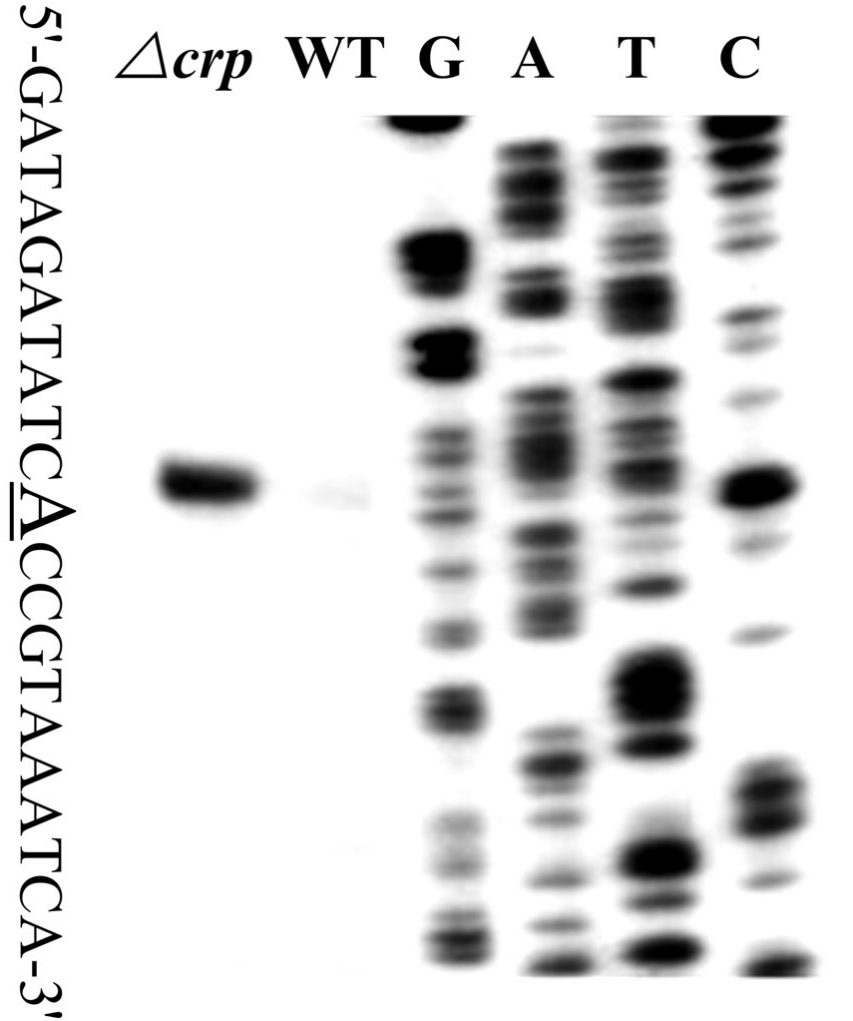
**Primer extension analysis**. Electrophoresis of the primer extension products was performed with a 6% polyacrylamide/8M urea gel. Lanes C, T, A and G represented the Sanger sequencing reactions. The transcriptional start sites were underlined.

The transcription start site of *sycO *was determined by primer extension assay. A single primer extension product was detected and thus a single CRP-dependent promoter was transcribed for *sycO-ypkA-yopJ *(Fig. 5). Compared to the WT, a much stronger primer extension product was detected in the *Δcrp*. Since the yield of primer extension product would indicate the mRNA expression level of *sycO *in each strain, data presented here confirmed the repression of *sycO-ypkA-yopJ *by CRP.

**Figure 6:**
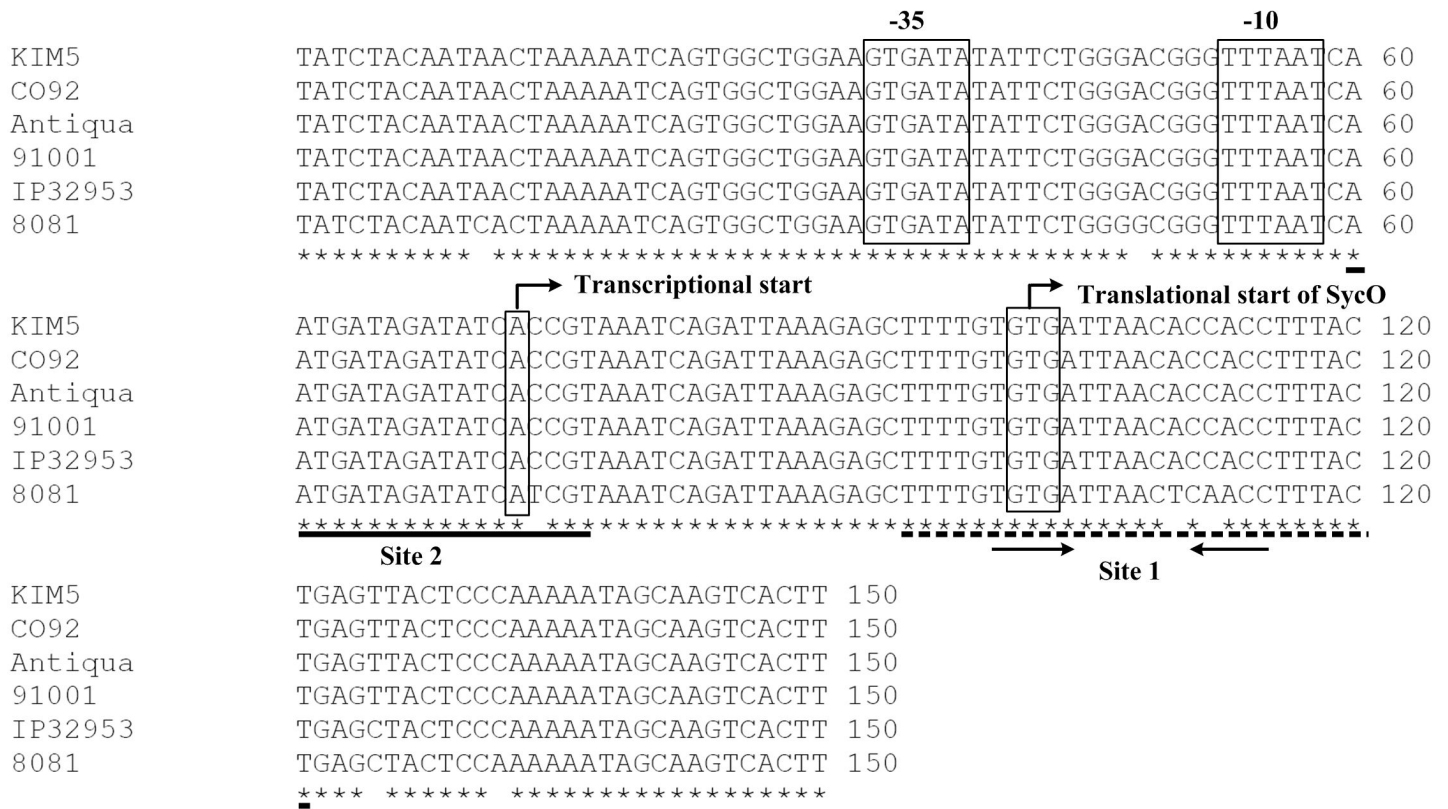
**Structural organization of the *sycO-ypkA-yopJ *promoter region**. The *sycO-ypkA-yopJ *promoter-proximate sequences (100 bp upstream to 50 bp downstream the start codon of *sycO*) from *Y. pestis *Antiqua (biovar *Antiqua*), KIM5 (*Mediaevalis*), CO92 (*Orientalis*) and 91001(*Microtus*), as well as those from *Y. pseudotuberculosis *IP32953 and *Y. enterocolitica *8081, were aligned and conserved nucleotide sites were labeled with asterisks. The CRP binding sites were underlined, and the invert repeats in the CPR box was showed with two invert arrows. Showed also were transcriptional/transcriptional start sites and promoter -10 and/or -35 elements.

The primer extension results could be also employed to map the 5' terminus of RNA transcript for *sycO *(i.e. the transcription start site of *sycO-ypkA-yopJ*) (Fig. 6). The -10 and -35 core promoter elements were predicted accordingly.

The determination of CRP-binding sites, transcription start site, and core promoter element (-10 and -35 regions) promoted us to depict the structural organization of CRP-dependent promoter, giving a map of CRP-promoter DNA interaction for *sycO-ypkA-yopJ *(Fig. 6).

## Discussion

### CRP and the *sycO-ypkA-yopJ *operon

CRP specifically bound to the *sycO *promoter-proximate region and directly repressed the expression of *sycO-ypkA-yopJ *in *Y. pestis *biovar *Microtus *strain 201. The *sycO-ypkA-yopJ *promoter-proximate regions were extremely conserved in *Y. pestis *(including all the four biovars *Antiqua *[21], *Mediaevalis *[22], *Orientalis *[20] and *Microtus *[19]), *Y. pseudotuberculosis *[23] and *Y. enterocolitica *[24]. Therefore, data presented in *Y. pestis *biovar *Microtus *can be generally applied to the above three pathogenic yersiniae.

A single CRP-dependent promoter transcribed for the *sycO-ypkA-yopJ *operon, but two CRP-binding sites (site 1 and site 2) were detected within its promoter region. A CRP box-like sequence (TAGATATCACC) was found in site 1 rather than in site 2. It was speculated that site 2 was a non-specific or non-functional CRP-binding site. Further reporter fusion experiments and/or *in vitr*o transcription assays, using the *sycO *promoter-proximate regions with different mutations/deletions within sites 1 and 2, should be done to elucidate the roles of site 1 and site 2 in CRP-mediated regulation of *sycO-ypkA-yopJ*.

### CRP and T3SS

The *crp *mutation caused a reduced secretion of YOP proteins in both *Y. enterocolitica *[5] and *Y. pestis *[9] grown under calcium-depleted conditions. This indicated that CRP is a positive regulator for the YOP secretion by *Y. pestis*. It is well known that the YOP secretion phenotype is only observable under calcium depleted conditions. Herein, the direct and negative regulation of *sycO-ypkA-yopJ *by CRP was observed at transcriptional level under calcium-rich conditions. How CRP controls T3SS is essentially unclear yet. It needs to investigate the mRNA/protein pools of T3SS that are regulated by CRP under calcium depleted or rich conditions and upon cell contact, and to answer whether CRP has a regulatory action on T3SS in general or on SycO, YpkA and YopJ specifically.

### CRP and virulence

The *crp *deletion attenuated *Y. pestis *much more greatly by subcutaneous route of infection in relative to an intravenous inoculation, and a reduced *in vivo *growth phenotype of the *crp *mutant was observed [4]. CRP seemed more important for the infection at the subcutaneous site and in the lymph other than the later systemic infection, while the reduced *in vivo *growth of the *crp *mutant should contribute to its attenuation by intravenous infection. The *crp *disruption led to a great defect of *pla *expression [4]. Since Pla specifically promoted *Y. pestis *dissemination from peripheral infection routes, the defect of *pla *expression in the *crp *mutant will contribute to the huge loss of virulence of this mutant strain after subcutaneous infection.

Expression of Pla, Pst, F1 antigen and T3SS are dependent on CRP, and this regulator appears to control a wide set of virulence-related factors in *Y. pestis *[4]. All the above CRP-regulated genes are harbored in plasmids that are required through horizontal gene transfer. Either the CRP protein itself or the mechanism of CRP-promoter DNA association is extremely conserved between *E. coli *and *Y. pestis*. Therefore, the above laterally acquired genes have evolved to integrate themselves into the 'ancestral' CRP regulatory cascade. It has been shown recently that the histone-like protein H-NS mediates the silencing of laterally acquired genes with low G+C contents scattered on the bacterial genome (these H-NS-dependent genes often contribute to virulence or host adaptation in corresponding pathogens) [25,26]. Herein, regulation (either activation or repression) of foreign genes in plasmids was mediated by the ancient regulator CRP in the host, *Y. pestis*.

## Conclusion

Three T3SS genes, *sycO*, *ypkA *and *yopJ*, constitute a single operon in *Y. pestis*. The CRP regulator binds to the upstream DNA region of *sycO*, and represses the expression of the *sycO-ypkA-yopJ *operon. The *sycO *promoter-proximate regions are extremely conserved in *Y. pestis*, *Y. pseudotuberculosis *and *Y. enterocolitica*, indicating that the CRP-dependent expression of *sycO-ypkA-yopJ *can be generally applied to the above three pathogenic yersiniae.

## Authors' contributions

DZ and RY conceived the study and designed the experiments. LJZ and LY performed all the experiments. LZ, YL and HG contributed to RT-PCR, primer extension assay and DNA binding assays. ZG participated in protein expression and purification. DZ, LFZ, CQ and DZ assisted in computational analysis and figure construction. The manuscript was written by LJZ and DZ, and revised by RY. All the authors read and approved the final manuscript.

## References

[B1] Ramamurthi KS Schneewind O Type iii protein secretion in yersinia species Annu Rev Cell Dev Biol 2002 18 107 133 10.1146/annurev.cellbio.18.012502.105912 12142275

[B2] Trosky JE Liverman AD Orth K Yersinia outer proteins: Yops Cell Microbiol 2008 10 3 557 565 10.1111/j.1462-5822.2007.01109.x 18081726

[B3] Zheng D Constantinidou C Hobman JL Minchin SD Identification of the CRP regulon using in vitro and in vivo transcriptional profiling Nucleic Acids Res 2004 32 19 5874 5893 10.1093/nar/gkh908 15520470 PMC528793

[B4] Zhan L Han Y Yang L Geng J Li Y Gao H Guo Z Fan W Li G Zhang L The cyclic AMP receptor protein, CRP, is required for both virulence and expression of the minimal CRP regulon in Yersinia pestis biovar microtus Infect Immun 2008 76 11 5028 5037 10.1128/IAI.00370-08 18710863 PMC2573370

[B5] Petersen S Young GM Essential role for cyclic AMP and its receptor protein in Yersinia enterocolitica virulence Infect Immun 2002 70 7 3665 3672 10.1128/IAI.70.7.3665-3672.2002 12065508 PMC128101

[B6] Oh MH Lee SM Lee DH Choi SH Regulation of the Vibrio vulnificus hupA gene by temperature alteration and cyclic AMP receptor protein and evaluation of its role in virulence Infect Immun 2009 77 3 1208 1215 10.1128/IAI.01006-08 19139193 PMC2643628

[B7] Skorupski K Taylor RK Cyclic AMP and its receptor protein negatively regulate the coordinate expression of cholera toxin and toxin-coregulated pilus in Vibrio cholerae Proc Natl Acad Sci USA 1997 94 1 265 270 10.1073/pnas.94.1.265 8990197 PMC19310

[B8] Rickman Lisa Scott Colin Debbie Hunt M Hutchinson Thomas Menendez M Carmen Whalan Rachael Hinds Jason Colston M Joseph Green J Buxton RS A member of the cAMP receptor protein family of transcription regulators in Mycobacterium tuberculosis is required for virulence in mice and controls transcription of the rpfA gene coding for a resuscitation promoting factor Molecular Microbiology 2005 56 5 1274 1286 10.1111/j.1365-2958.2005.04609.x 15882420 PMC2964915

[B9] Kim TJ Chauhan S Motin VL Goh EB Igo MM Young GM Direct transcriptional control of the plasminogen activator gene of Yersinia pestis by the cyclic AMP receptor protein J Bacteriol 2007 189 24 8890 8900 10.1128/JB.00972-07 17933899 PMC2168602

[B10] Sebbane F Jarrett CO Gardner D Long D Hinnebusch BJ Role of the Yersinia pestis plasminogen activator in the incidence of distinct septicemic and bubonic forms of flea-borne plague Proceedings of the National Academy of Sciences of the United States of America 2006 103 14 5526 5530 10.1073/pnas.0509544103 16567636 PMC1414629

[B11] Lathem WW Price PA Miller VL Goldman WE A plasminogen-activating protease specifically controls the development of primary pneumonic plague Science 2007 315 5811 509 513 10.1126/science.1137195 17255510

[B12] Park H Teja K O'Shea JJ Siegel RM The Yersinia effector protein YpkA induces apoptosis independently of actin depolymerization J Immunol 2007 178 10 6426 6434 17475872 10.4049/jimmunol.178.10.6426

[B13] Mukherjee S Keitany G Li Y Wang Y Ball HL Goldsmith EJ Orth K Yersinia YopJ acetylates and inhibits kinase activation by blocking phosphorylation Science 2006 312 5777 1211 1214 10.1126/science.1126867 16728640

[B14] Viboud GI Bliska JB YERSINIA OUTER PROTEINS: Role in Modulation of Host Cell Signaling Responses and Pathogenesis Annu Rev Microbiol 2005 59 69 89 10.1146/annurev.micro.59.030804.121320 15847602

[B15] Dittmann S Schmid A Richter S Trulzsch K Heesemann J Wilharm G The Yersinia enterocolitica type three secretion chaperone SycO is integrated into the Yop regulatory network and binds to the Yop secretion protein YscM1 BMC Microbiol 2007 7 67 10.1186/1471-2180-7-67 17612396 PMC1933539

[B16] Zhou D Tong Z Song Y Han Y Pei D Pang X Zhai J Li M Cui B Qi Z Genetics of metabolic variations between Yersinia pestis biovars and the proposal of a new biovar, microtus J Bacteriol 2004 186 15 5147 5152 10.1128/JB.186.15.5147-5152.2004 15262951 PMC451627

[B17] Datsenko KA Wanner BL One-step inactivation of chromosomal genes in Escherichia coli K-12 using PCR products Proc Natl Acad Sci USA 2000 97 12 6640 6645 10.1073/pnas.120163297 10829079 PMC18686

[B18] Straley SC Bowmer WS Virulence genes regulated at the transcriptional level by Ca2+ in Yersinia pestis include structural genes for outer membrane proteins Infect Immun 1986 51 2 445 454 3002984 10.1128/iai.51.2.445-454.1986 PMC262351

[B19] Song Y Tong Z Wang J Wang L Guo Z Han Y Zhang J Pei D Zhou D Qin H Complete genome sequence of Yersinia pestis strain 9 an isolate avirulent to humans DNA Res 1001 11 3 179 197 10.1093/dnares/11.3.179 15368893

[B20] Parkhill J Wren BW Thomson NR Titball RW Holden MT Prentice MB Sebaihia M James KD Churcher C Mungall KL Genome sequence of Yersinia pestis, the causative agent of plague Nature 2001 413 6855 523 527 10.1038/35097083 11586360

[B21] Chain PS Hu P Malfatti SA Radnedge L Larimer F Vergez LM Worsham P Chu MC Andersen GL Complete genome sequence of Yersinia pestis strains Antiqua and Nepal516: evidence of gene reduction in an emerging pathogen Journal of bacteriology 2006 188 12 4453 4463 10.1128/JB.00124-06 16740952 PMC1482938

[B22] Deng W Burland V Plunkett G 3rd Boutin A Mayhew GF Liss P Perna NT Rose DJ Mau B Zhou S Genome sequence of Yersinia pestis KIM J Bacteriol 2002 184 16 4601 4611 10.1128/JB.184.16.4601-4611.2002 12142430 PMC135232

[B23] Chain PS Carniel E Larimer FW Lamerdin J Stoutland PO Regala WM Georgescu AM Vergez LM Land ML Motin VL Insights into the evolution of Yersinia pestis through whole-genome comparison with Yersinia pseudotuberculosis Proc Natl Acad Sci USA 2004 101 38 13826 13831 10.1073/pnas.0404012101 15358858 PMC518763

[B24] Thomson NR Howard S Wren BW Holden MT Crossman L Challis GL Churcher C Mungall K Brooks K Chillingworth T The complete genome sequence and comparative genome analysis of the high pathogenicity Yersinia enterocolitica strain 8081 PLoS Genet 2006 2 12 e206 10.1371/journal.pgen.0020206 17173484 PMC1698947

[B25] Lucchini S Rowley G Goldberg MD Hurd D Harrison M Hinton JC H-NS mediates the silencing of laterally acquired genes in bacteria PLoS Pathog 2006 2 8 e81 10.1371/journal.ppat.0020081 16933988 PMC1550270

[B26] Navarre WW Porwollik S Wang Y McClelland M Rosen H Libby SJ Fang FC Selective silencing of foreign DNA with low GC content by the H-NS protein in Salmonella Science 2006 313 5784 236 238 10.1126/science.1128794 16763111

